# Enhancing newborn screening sensitivity and specificity for missed NICCD using selected amino acids and acylcarnitines

**DOI:** 10.1186/s13023-025-03532-7

**Published:** 2025-01-11

**Authors:** Peiyao Wang, Lingwei Hu, Yuhe Chen, Duo Zhou, Shasha Zhu, Ting Zhang, Ziyan Cen, Qimin He, Benqing Wu, Xinwen Huang

**Affiliations:** 1 Present Address: Department of Genetics and Metabolism, Children’s Hospital of Zhejiang University School of Medicine, National Clinical Research Center for Child Health, No. 3333 Binsheng Road, Binjiang District, Hangzhou, 310053 Zhejiang China; 2 Department of Pediatric Health, Taizhou Women and Children’s Hospital, Taizhou, 318000 Zhejiang China; 3 School of Geography Science and Geomatics Engineering, Suzhou University of Science and Technology, Suzhou, 215009 Jiangsu China; 4 Children’s Medical Center, University of the Chinese Academy of Sciences-Shenzhen Hospital, Shenzhen, 518106 Guangdong China

**Keywords:** NICCD, Missed screening, Citrulline, Newborn screening, *SLC25A13*, Dried blood spots

## Abstract

**Purpose:**

To enhance the detection rate of Neonatal Intrahepatic Cholestasis caused by Citrin Deficiency (NICCD) through newborn screening (NBS), we analyzed the metabolic profiles of missed patients and proposed a more reliable method for early diagnosis.

**Methods:**

In this retrospective study, NICCD patients were classified into “Newborn Screening” (64 individuals) and “Missed Screening” (52 individuals) groups. Metabolic profiles were analyzed using the non-derivatized MS/MS Kit, and genetic mutations were identified via next-generation sequencing and confirmed by Sanger sequencing. Receiver Operating Characteristic (ROC) analysis evaluated the predictive value of amino acids and acylcarnitines in dried blood spots (DBS) for identifying missed patients including 40 missed patients and 17,269 healthy individuals, with additional validation using 12 missed patients and 454 healthy controls.

**Results:**

The age of diagnosis was significantly higher in the “Missed Screening” group compared to the “Newborn Screening” group (74.50 vs. 18.00 days, *P* < 0.001). ROC analysis revealed that citrulline had excellent diagnostic accuracy for missed patients, with an AUC of 0.970 and a cut-off value of 17.57 µmol/L. Additionally, glycine, phenylalanine, ornithine, and C8 were significant markers, each with an AUC greater than 0.70. A combination of these markers achieved an AUC of 0.996 with a cut-off value of 0.00195. Validation demonstrated a true positive rate of 91.67% and a true negative rate of 96.48%. Common *SLC25A13* mutations in both groups were c.852_855del, IVS16ins3kb, and c.615 + 5G > A.

**Conclusions:**

Combining multiple metabolic markers during NBS significantly improves sensitivity and specificity for detecting missed NICCD cases. However, the relationship between genetic mutations and missed cases remains unclear.

**Supplementary Information:**

The online version contains supplementary material available at 10.1186/s13023-025-03532-7.

## Introduction

Neonatal Intrahepatic Cholestasis caused by Citrin Deficiency (NICCD) is one of the most common inherited metabolic disorders in East Asia and a primary cause of intrahepatic cholestasis in infants. NICCD is typically diagnosed following elevated citrulline (Cit) levels during newborn screening (NBS). However, some children do not exhibit elevated Cit levels at birth but develop cholestasis within two to three months. The carrier rate of pathogenic variants in the *SLC25A13* gene in China is 1/65, leading to an estimated disease prevalence of 1/17,000 [1]. However, the actual incidence of citrin deficiency (CD) detected via NBS nationwide is only 1/68,000 [2], and in Zhejiang Province, it is as low as 1/82,352[3]. The Zhejiang Neonatal Disease Screening Center, one of the largest centers for genetic metabolic disorders in China, has focused on collecting missed NICCD over the past decade. Despite this effort, only a few dozen cases have been clinically detected, suggesting that a significant number of missed patients remain undiagnosed. Some of these patients may still be asymptomatic, while a small fraction could progress to cirrhosis and liver failure, necessitating liver transplantation [4]. Research indicates that later-onset patients have a worse prognosis compared to those identified early [5]. Therefore, early identification, diagnosis, and treatment are critically important. However, since Cit levels during NBS in both missed screening and healthy newborns are within the normal range, identifying missed patients from tandem mass spectrometry (MS/MS) is challenging.

Citrin, an aspartate-glutamate carrier (AGC) primarily expressed in the liver, is a component of the malate-aspartate shuttle[6]. AGC plays a crucial role in transporting aspartate into the cytoplasm and glutamate into the mitochondria, indirectly facilitating NADH transfer from the cytoplasm to the mitochondria. This balance is necessary for the synthesis of urea, proteins, and nucleotides[7]. Citrin deficiency disrupts various metabolic pathways, including the urea cycle, aerobic glycolysis, gluconeogenesis, galactose metabolism, and fatty acid synthesis [8–10]. These disruptions cause biochemical abnormalities such as elevated transaminases, increased bilirubin, and hypercholesterolemia, as well as metabolic abnormalities like elevated levels of multiple amino acids, including Cit with or without methionine (Met), phenylalanine (Phe), tyrosine (Tyr), and ornithine (Orn), along with abnormal acylcarnitine profiles [11–14]. However, current research primarily focuses on NICCD cases with evident metabolic abnormalities, while studies on the metabolic profiles of missed cases during NBS are limited. Thus, it remains unclear whether certain metabolic indicators, which fall within normal ranges yet differ from those of healthy newborns, could serve as early markers for identifying these missed cases.

CD is a hereditary metabolic disorder caused by autosomal recessive mutations in the *SLC25A13* gene [15]. Four major mutations account for almost 80% of pathogenic alleles in the Chinese population [16]. These mutations exhibit geographic distribution differences [17]. Beyond NICCD, CD presents with two additional age-dependent clinical phenotypes: Failure to Thrive and Dyslipidemia caused by Citrin Deficiency (FTTDCD), which occurs in older children and is characterized by growth retardation and abnormal blood lipids; and Adult Onset Type II Citrullinemia (CTLN2), which manifests in adolescence or adulthood [18–20]. However, genotype-phenotype correlations in CD remain unclear, and there is no definite relationship between Cit concentration and genotype [21]. Therefore, we aim to investigate the genetic distribution in both NBS-positive and missed patients to determine if there is a correlation between specific gene mutations and phenotype.

To enhance the positive detection rate of NICCD in NBS, we identified multiple metabolic indicators to detect missed patients and analyzed the genetic distribution of both missed and NBS-positive patients.

## Methods

### Setting

We conducted a single-center retrospective study at the Children's Hospital, Zhejiang University School of Medicine. This study included patients diagnosed with NICCD for the first time in the Department of Genetic Metabolism or Gastroenterology between January 2011 and September 2023. The Ethical Committee of Children’s Hospital, Zhejiang University School of Medicine, approved this study, and written consents were obtained from parents for sample collection and data publication.

### Study design and data collection

**Fig. 1:**
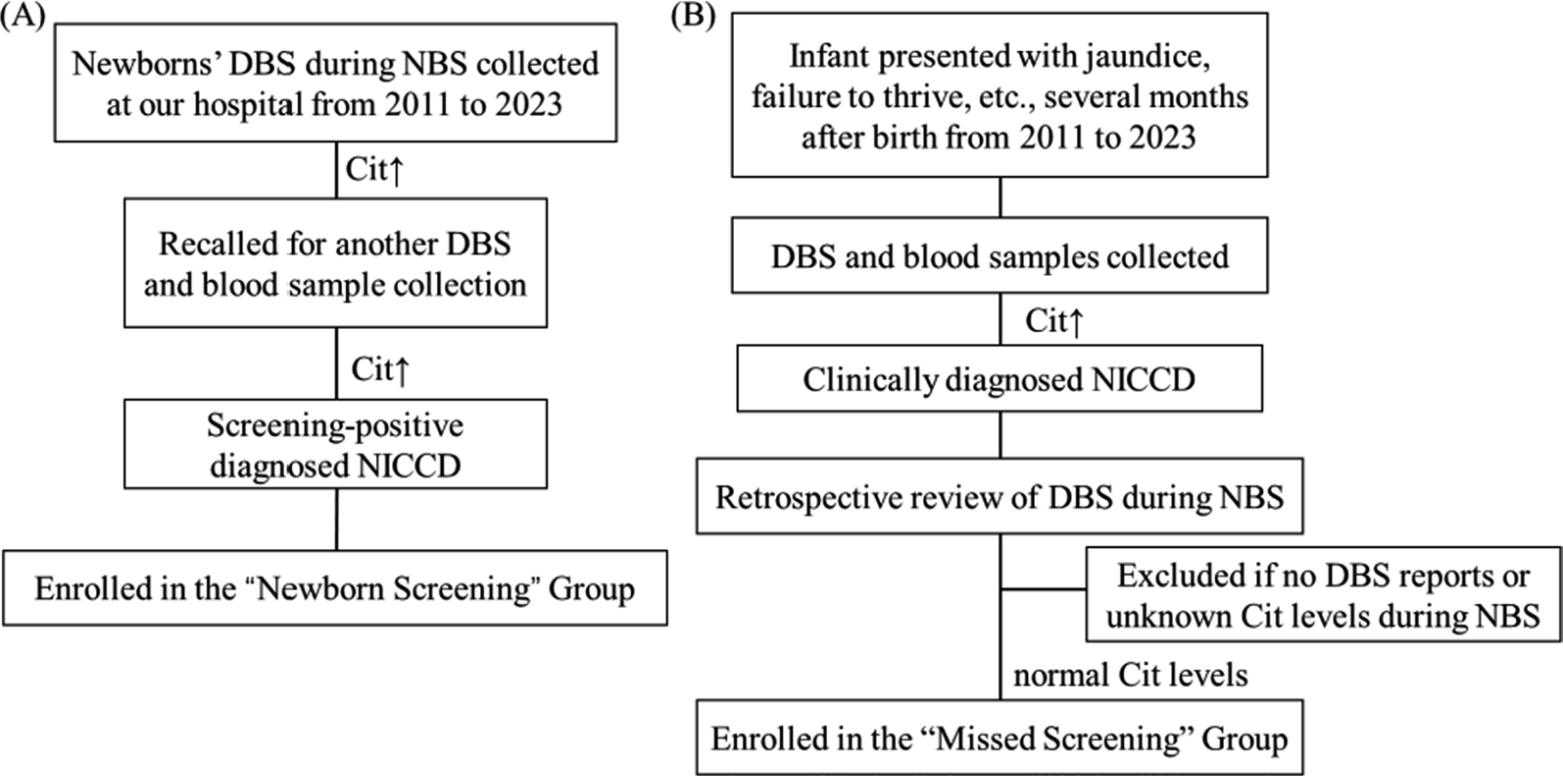
Flow diagram

Our NBS protocol aligns with the standard practices followed by screening centers both in China and internationally. During NBS, some newborns showed elevated Cit concentrations in dried blood spots (DBS). They were recalled for another DBS testing and blood sample collection. If their Cit levels remained elevated and were confirmed genetically as NICCD, along with biochemical indicators, they were classified into the “Newborn Screening” group, which included 64 individuals (Fig. 1A). Additionally, some patients were admitted to the hospital with symptoms such as prolonged jaundice and failure to thrive. Upon admission, DBS and blood samples revealed elevated Cit levels, bilirubin, or liver enzymes, clinically suggesting neonatal intrahepatic cholestasis. A retrospective review of these patients' DBS results during NBS was conducted. Patients with normal Cit levels during NBS were classified into the “Missed Screening” group while those with unavailable or unclear Cit levels were excluded. This group included 52 individuals (Fig. 1B). Sociodemographic information and metabolic indices during NBS were collected from electronic medical records.

### ROC analysis for predicting missed screening patients

We used Receiver Operating Characteristic (ROC) analysis to evaluate the predictive value of amino acids, free carnitines, and acylcarnitines during NBS for identifying missed NICCD patients. Using MedCalc, we estimated the required sample size for the healthy control group, setting α = 0.05, Power = 0.95, Area under ROC curve = 0.7, Null Hypothesis value = 0.5, and the ratio of sample sizes in negative/positive groups = 500. The estimated sample size was 28 missed screening patients and 14,000 healthy individuals. Ultimately, we included 40 missed screening patients and 17,269 age- and sex-matched healthy individuals, all born in Zhejiang Province, in the ROC analysis. The remaining 12 missed screening patients and an additional 454 healthy controls were used to validate the ROC results.

### Metabolic index detection and molecular testing

The DBS samples collected during NBS were obtained 3–7 days after birth using the heel prick method and then spotted onto the Whatman 903 filter paper. Amino acid and acylcarnitine profiles were measured by the NeoBase non-derivatized MS/MS Kit (PerkinElmer, Finland). The procedure involved adding 100 μl of a working solution containing an internal standard to a U-bottom plate, vibrating at 650 rpm, and incubating at 45 °C for 45 min. Then, 75 μl of the liquid was transferred to a V-bottom plate and incubated at room temperature for 2 h before injecting 25 μl into MS/MS for metabolic analysis. Quality control includes low and high-level internal quality controls [22]. MS/MS results were used to compare the metabolic characteristics of amino acids and acylcarnitines among different groups and to construct predictive models.

### Detection of mutation

Genomic DNA was extracted from blood samples of the proband and their parents. Liquid-phase capture technology targeted 166 genes related to commonly inherited metabolic diseases, followed by next-generation sequencing and bioinformatics analysis. Suspected variants were confirmed by Sanger sequencing in the proband and family members, followed by genetic interpretation.

### Treatment

Both groups received the same treatment regimen for NICCD, which includes: (1) Dietary therapy with lactose-free, medium-chain triglyceride formula milk; (2) Symptomatic treatment including liver protection treatment and arginine to reduce ammonia levels; (3) Supplementation of fat-soluble vitamins and other micronutrients; and (4) Introduction of a low-carbohydrate and high-fat, high-protein diet after complementary foods were added. All data in this study were collected before treatment began, ensuring that treatment had no impact on the results.

### Statistical analysis

SPSS 26.0 software was used for statistical analysis of metabolic indices. Continuous variables were described as mean ± standard deviation (SD). Continuous variables with a normal distribution were compared using Student's t-test, while variables with a non-normal distribution were compared using the Mann-Whitney or Kruskal-Wallis. Categorical variables were analyzed using the chi-square test. P < 0.05 was considered statistically significant. An AUC of the ROC curve between 0.70 and 0.90 indicates medium accuracy, and an AUC > 0.90 indicates excellent test accuracy [23]. Combined indicators from the ROC analysis were used for binary logistic regression. GraphPad Prism 8 software was used to draw gene distribution charts. The structure stability analysis of the novel missense variant was performed using Chimera ver 1.17.3.

## Results

### Sociodemographic of the “newborn screening” and “missed screening” group

Table 1 shows that the age of diagnosis in the “Missed Screening” group was 74.50 (58.75,108.00) days, significantly greater than the “Newborn Screening” group 18.00 (14.00,21.00) days (P < 0.001). There were no statistically significant differences between the two groups in terms of gender, birth weight, and gestational age (P > 0.05).

**Table 1:** Sociodemographic information of the “Newborn Screening” and “Missed Screening” group

Variables	Newborn Screening Group, N = 64	Missed Screening Group, N = 52	*χ*^*2*^/t/Z	*P* value
Gender			0.008	0.93
Male	45.31%(n = 29)	46.15%(n = 24)		
Female	54.69%(n = 35)	52.83%(n = 28)		
Birth weight (kg)	2.74 ± 0.51	2.87 ± 0.44	−1.35	0.18
Gestational age (weeks)	38.00(38.00,39.75)	39.00(38.00,39.75)	−0.92	0.36
Diagnostic age (days)	18.00(14.00,21.00)	74.50(58.75,108.00)	−8.43	< 0.001

### Comparison of various amino acids and acylcarnitines during NBS among three groups

We compared indicators including amino acids and acylcarnitines during NBS between healthy control, “Newborn Screening” and “Missed Screening” groups. In the “Newborn Screening” group, indicators with an abnormal rate (proportion of values exceeding or falling below the normal range) greater than 10% included Cit, Met, Phe, Tyr, Arg, and Gly. Among these, Cit, Met, Phe, Tyr, and Arg were elevated compared to the healthy control group, while Gly was lower. Some additional metabolites were significantly different between the two groups but remained within the reference range. Although the abnormal rates of all amino acid and acylcarnitine indicators in the “Missed Screening” and healthy control group are within 10%, slight abnormalities were observed in some indicators between the two groups. Cit, Arg, Orn, Pro, Phe, Gly, C3, C6, C8, C16, and C18 showed statistically significant differences compared to the control group (P < 0.05). These indicators are considered potential markers for missed NICCD (Table 2).

**Table 2:** Comparison of amino acids and acylcarnitines during NBS among three groups

	Reference intervals (µmol/L)	Healthy controls medians (interquartile range) (N = 17,269)	“Missed Screening” group medians (interquartile range) (N = 52)	“Newborn Screening” group medians (interquartile range) (N = 64)	P (Three group)	Missed vs control	NBS vs control
Cit	7.9–37	13.45(11.46,15.6)	25.70(19.66,30.03)	**97.06(69.65,230.27)**	< 0.001	< 0.001	< 0.001
Phe	23.3–100	60.16(53.07,68.44)	49.61(43.79,55.81)	**81.48(57.22,119.57)**	< 0.001	< 0.001	< 0.001
Met	7.18–41.35	16.22(13.79,19.11)	17.99(13.13,21.77)	**28.43(20.88,43.93)**	< 0.001	0.79	< 0.001
Tyr	34.5–250	114.65(91.35,146.40)	108.35(81.66,131.11)	**195.27(116.81,278.47)**	< 0.001	0.14	< 0.001
Ala	136.5–650	310.28(258.41,376.42)	335.66(287.14,382.90)	268.66(212.78,376.52)	< 0.01	0.24	< 0.05
Leu	75.7–316	168.12(145.53,194.81)	159.90(140.85,185.40)	178.95(147.61,212.76)	0.07	–	–
Arg	2.54–50	5.11(2.90,8.57)	10.46(4.86,16.01)	**17.69(10.48,32.32)**	< 0.001	< 0.001	< 0.001
Orn	52.09–323.22	121.19(99.84,148.66)	150.38(129.38,183.34)	162.13(126.14,196.26)	< 0.001	< 0.001	< 0.001
Gly	246.57–1283	535.32(458.21,627.35)	390.75(341.06,478.95)	**352.38(280.47,463.63)**	< 0.001	< 0.001	< 0.001
Val	51.7–270	142.95(124.65,164.20)	136.78(122.58,163.71)	177.43(145.72,214.37)	< 0.001	1	< 0.001
Pro	97.2–401.5	200.51(173.54,234.40)	241.32(195.52,298.85)	223.81(184.71,270.75)	< 0.001	< 0.001	< 0.01
C0	10.28–54.24	24.49(20.00,30.16)	23.86(18.89,28.87)	27.72(22.79,39.14)	< 0.001	0.83	< 0.001
C2	3–50	17.51(13.97,21.82)	18.53(15.39,22.46)	16.29(12.46,24.12)	0.39	–	–
C3	0.43–3.8	1.57(1.23,2.04)	1.42(1.17,1.72)	1.71(1.28,2.33)	< 0.05	< 0.05	0.30
C4	0.03–0.48	0.21(0.17,0.26)	0.22(0.17,0.26)	0.23(0.19,0.31)	0.06	–	–
C5	0.01–0.4	0.10(0.08,0.13)	0.11(0.08,0.12)	0.14(0.12,0.20)	< 0.001	1	< 0.001
C6	0.03–0.17	0.04(0.03,0.05)	0.05(0.08,0.10)	0.04(0.03,0.06)	< 0.001	< 0.001	< 0.05
C8	0.02–0.17	0.05(0.04,0.07)	0.08(0.05,0.10)	0.05(0.04,0.08)	< 0.001	< 0.001	1
C10	0.03–0.22	0.07(0.06,0.10)	0.09(0.06,0.13)	0.05(0.04,0.78)	< 0.001	0.07	< 0.001
C12	0.03–0.28	0.08(0.06,0.11)	0.09(0.06,0.13)	0.06(0.04,0.10)	< 0.001	0.18	< 0.001
C14	0.07–0.4	0.19(0.15,0.23)	0.18(0.15,0.23)	0.18(0.14,0.24)	0.55	–	–
C16	0.49–6	3.22(2.50,4.05)	2.68(2.13,3.38)	2.15(1.39,3.33)	< 0.001	< 0.01	< 0.001
C18	0.24–1.79	0.88(0.71,1.07)	0.76(0.59,0.90)	0.75(0.52,0.92)	< 0.001	< 0.01	< 0.001

The bold type means the abnormal rate is higher than 10%

The data in the "Newborn Screening" group are from the first DBS during NBS

### The diagnostic value of amino acids and acylcarnitines for missed screening patients

Table 3 illustrates the diagnostic value of differential amino acids and acylcarnitines for detecting missed screening patients during NBS compared to a control group. The ROC curves for Cit exhibited excellent accuracy with an AUC of 0.970. In comparison, the ROC curves for Gly, Phe, Orn, and C8 demonstrated moderate accuracy, with AUC values of 0.806, 0.762, 0.703, and 0.729, respectively. The ROC curves for Pro, Arg, C6, C16, C18, and C3 showed lower accuracy, all with AUC values below 0.70. The cut-off levels of these indicators are Cit (17.57 μmol/L), Gly (480.5 μmol/L), Phe (51.36 μmol/L), Orn (126.8 μmol/L), and C8 (0.07 μmol/L). Additionally, the combination of Cit, Gly, Phe, Orn, and C8, which all had an AUC > 0.70, significantly enhances diagnostic accuracy, achieving an AUC of 0.996, sensitivity of 97.5%, and specificity of 97.4% with a cut-off level of 0.00195.

**Table 3:** The diagnostic value of DBS amino acids and acylcarnitines for missed patients

	Cut-off(µmol/L)	AUC	*P* value	Sensitivity (%)	Specificity (%)
**Cit**	17.57	0.970	< .001	95	91.67
**Gly**	480.5	0.806	< .001	80	67.72
**Phe**	51.36	0.762	< .001	65	80.29
**Orn**	126.8	0.703	< .001	77.5	55.95
Pro	230.4	0.680	< .001	60	72.7
Arg	9.97	0.672	< .001	55	80.96
**C8**	0.07	0.729	< .001	72.5	72.77
C6	0.05	0.676	< .001	65	68.24
C16	2.87	0.670	< .001	67.5	62.88
C18	0.86	0.658	< .001	75	53.3
C3	1.55	0.628	< .01	72.5	52.05
A combination of Cit, Orn, Phe, Gly, C8	0.00195	0.996	< .001	97.5	97.4

The bold type means AUC > 0.70

### Prediction and validation of missed NICCD Patients using Cit, Gly, Orn, Phe, and C8

A binary logistic regression analysis of Cit, Gly, Orn, Phe, and C8 (Table 4), resulted in the following regression equation:

**Table 4:** Binary logistic regression of predictor variables

	β	P	OR	95% Confidence Interval	
				Lower Bound	Upper Bound
Cit	1.15	< 0.001	3.17	2.10	4.80
Gly	−0.021	< 0.001	0.98	0.97	0.99
Phe	−0.120	< 0.01	0.89	0.82	0.96
Orn	0.044	< 0.001	1.05	1.02	1.07
C8 (expanded 100 times)*	0.345	< 0.001	1.41	1.23	1.63
Constant	−17.77	< 0.001	0	–	–

^*^We expanded the C8 variable by 100 times to address the extremely large ORs and CIs from the original variable

Total score = 1.155 × Cit-0.021 × Gly-0.12 × Phe + 0.044 × Orn + 34.5 × C8-17.77.

**Fig. 2:**
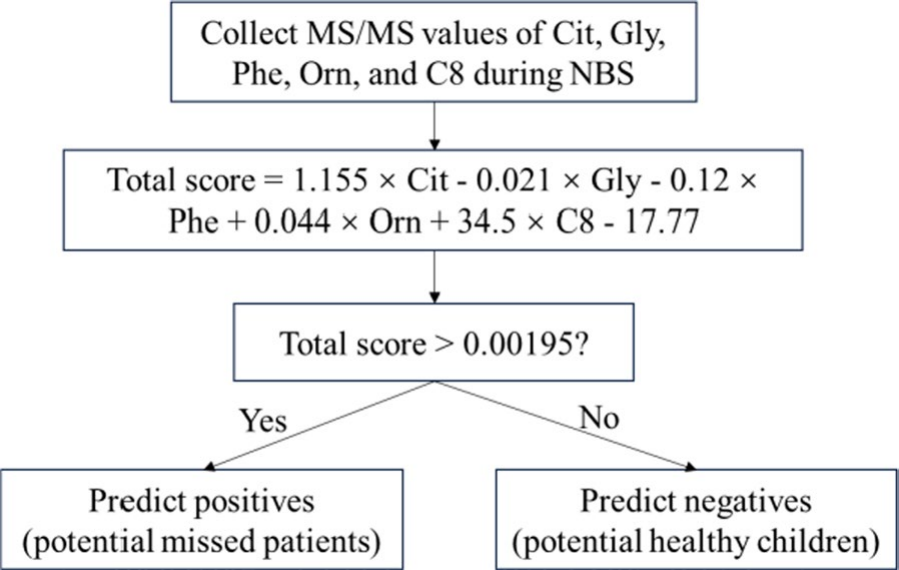
Flowchart for predicting using combined indicators' cut-off

**Fig. 3:**
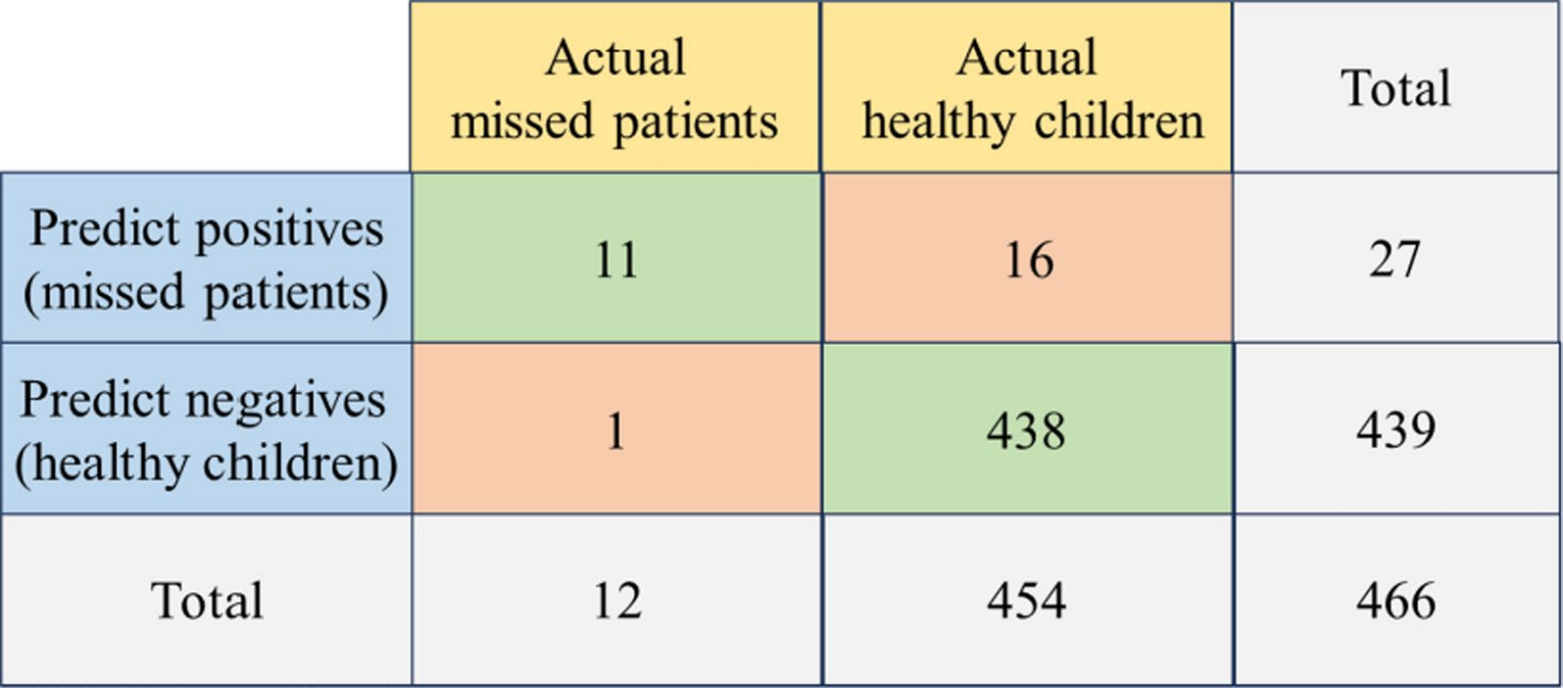
Validation with missed patients and healthy controls

To validate the model's predictive ability, we retrospectively collected MS/MS data during NBS from an additional 12 missed patients and 454 healthy children. We applied Cit, Gly, Orn, Phe, and C8 to this regression equation. If the total score exceeded the cut-off value of 0.00195, determined by the ROC curve for the combined variables, the prediction was classified as a missed patient; otherwise, it was classified as a healthy child (Fig. 2). The validation results demonstrated a True Positive rate (sensitivity) of 91.67% (11/12), a True Negative rate (specificity) of 96.48% (438/454), a False Positive rate of 3.52% (16/454), a False Negative rate of 8.33% (1/12), and a Positive Predictive Value (PPV) of 40.74% (11/27) (Fig. 3).

### Genetic findings

**Fig. 4:**
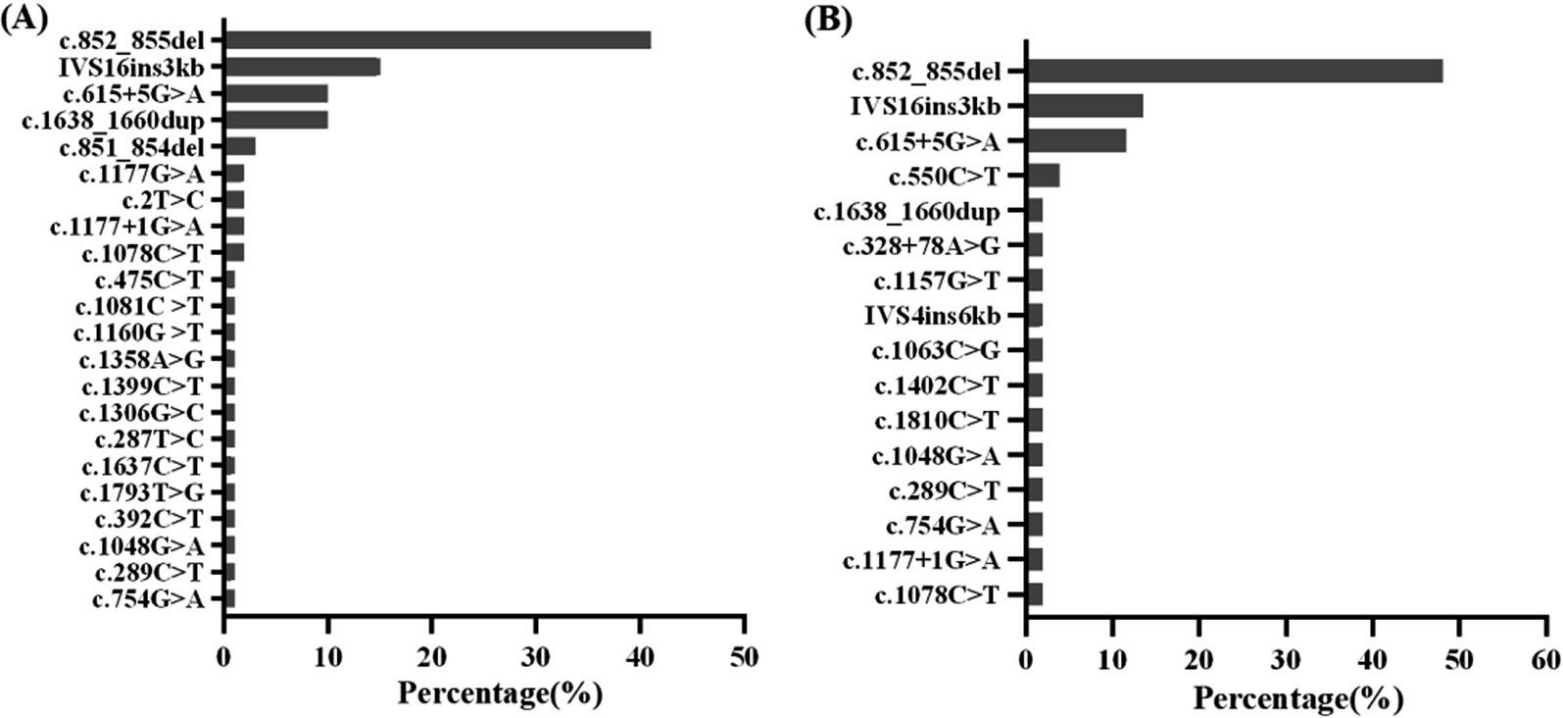
Mutation frequency in *SLC25A13* (NM_014251.3): Newborn Screening (A) vs. Missed Screening (B)

A total of 29 *SLC25A13*(NM_014251.3) mutations were detected, with 22 in the “Newborn Screening” group and 16 in the “Missed Screening” group. In both groups, c.852_855del, IVS16ins3kb, and c.615 + 5G > A were the most frequent mutations. In the “Newborn Screening” group, the mutation frequencies were 41%, 15%, and 10%, respectively, while in the “Missed Screening” group, they were 48.08%, 13.46%, and 11.54%, respectively (Fig. 4). The c.1638_1660dup mutation accounts for 10% in the “Newborn Screening” group but only 1.92% in the “Missed Screening” group. The *SLC25A13* mutations in both groups were primarily concentrated in these four mutations, accounting for 76% and 75%, respectively. A novel mutation c.392C > T was found in the “Newborn Screening” group and its protein model is presented in the supplement file.

## Discussion

Missed NICCD cases are flagged as normal during NBS because their Cit levels are below the screening cutoff, resulting in many false-negative results. To address this, we retrospectively identified differential metabolisms between missed cases and healthy controls, assessing their sensitivity and specificity. We validated these markers as predictors of missed NICCD and compared the genetic distribution between missed and NBS-positive patients.

Consistent with previous findings [24, 25], the onset age of the “Missed Screening” group was approximately 2-3 months, older than the “Newborn Screening” group. The Cit level of “Missed Screening” patients during NBS was normal, likely due to early blood sampling before metabolic profiles had fully manifested [26]. Late referral and failure to switch to a high-MCT or lactose-free formula were associated with poor prognosis in NICCD [27]. In later-onset symptomatic patients, fatty acid oxidation, liver function, and cholestasis were more severely impaired compared to those identified through NBS, as evidenced by higher transaminase, direct bilirubin, bile acids, dyslipidemia, and lower protein levels [3]. Among the missed patients in our study, two were not diagnosed with NICCD until they were 9-10 months old, at which point liver cirrhosis was detected, ultimately necessitating liver transplantation. These findings emphasize the importance of early diagnosis and management.

Citrin deficiency disrupts multiple metabolic pathways, leading to abnormalities in amino acids and acylcarnitines. The deficiency impairs the synthesis of argininosuccinate, resulting in the accumulation of intermediate products of the urea cycle such as Cit, Arg, and Orn [3]. Additionally, aromatic amino acids (AAA) like Phe, Tyr, and tryptophan, as well as Met, increase due to cholestatic liver injury [28]. Glycogenic amino acids like Gly are significantly reduced, which may indicate enhanced gluconeogenic activity. This is consistent with previous studies showing that impaired gluconeogenesis from lactate caused by an increased NADH/NAD+ ratio that inhibits the conversion of lactate to pyruvate[29], leads to greater reliance on glycogenic amino acids like glycine, serine, and alanine, ultimately resulting in their decreased levels[3, 26, 30].

The current Cit cutoff for screening NICCD is inadequate, as many missed patients have Cit levels below this threshold, resulting in numerous false negative results [31]. Using amino acids and acylcarnitines from established NBS systems to identify missed patients is a highly feasible, straightforward, and cost-effective method. Zhang et al. [3] found that Cit, Arg, Met, Orn, Phe, Ala, Leu, Val, C0, C3, C16:1OH, C18:1, C18:2, ammonia, aspartate transaminase, and total bile acids contributed most to the differentiation between the "newborn screening group" and the "clinical diagnosis group". Our study found Cit, Arg, Orn, Pro, Phe, Gly, C3, C6, C8, C16, and C18 to be potential differential markers for missed NICCD. Cit was the first abnormality detected after birth. To address the issue of false negatives, Chen et al. [32] used Cit levels > 20 μmol/L as the first tier and gene analysis as the second tier for detection. By simultaneously evaluating Cit and the Cit/tAA ratio, MS/MS demonstrates high sensitivity for detecting NICCD, identifying nearly 80% of previously missed patients [31]. Consistent with previous studies, Cit remained the best indicator with high sensitivity and specificity in our cohort. Specifically, a Cit value of 17.57 μmol/L was the optimal diagnostic cutoff for missed patients, approximately half of the upper limit (min-max: 7.14-37 μmol/L). This was also consistent with the hypothesis we previously formulated based on clinical experience [33]. In addition, Gly, Phe, Orn, and C8 also served as moderate accuracy indicators (AUC > 0.7) for identifying missed patients. Therefore, we propose a combined evaluation using Cit, Gly, Phe, Orn, and C8 levels (combined AUC = 0.996) to improve the detection rate for NICCD. This approach offers higher accuracy, sensitivity, and specificity, with a lower false positive rate compared to using a single amino acid or acylcarnitine.

Previous studies have developed a combined evaluation method for detecting newborns who later develop NICCD, which uses a scoring system based on five biomarkers (Arg, Cit, Ile + Leu, Tyr, and C0/C5-DC), where exceeding specific thresholds for these biomarkers results in a score; a total score of 4 or more indicates a high risk of developing NICCD [34]. Despite its high specificity (98.7%), it has a low sensitivity (66.7%), resulting in a high rate of missed diagnoses and limited predictive power. In our study, we developed a new predictive formula using binary logistic regression. This formula requires only the values for Cit, Gly, Phe, Orn, and C8 from the NBS, making it especially useful for large samples of 10,000 or more without additional steps or tests. Validation with 12 confirmed missed patients and 454 healthy children demonstrated high sensitivity and specificity, both exceeding 90%. This indicates that our formula is simple, efficient, and highly predictive. Our study achieved a false negative rate below 10% and a false positive rate below 5%. To further reduce false positives or false negatives, we propose combining this predictive method with a high-throughput iPLEX genotyping assay to detect *SLC25A13* mutations [33]. Additionally, our ongoing non-targeted metabolomics research on NICCD will offer new approaches for screening missed patients. Notably, the PPV in our study was relatively low. For rare diseases like NICCD, a lower PPV is expected, even with a highly sensitive and specific screening tool, because PPV is influenced by both test accuracy and the disease's prevalence. Therefore, the low PPV observed in this study is largely due to the low prevalence of confirmed missed NICCD cases in our sample.

Delayed diagnosis and treatment of NICCD imposes a significant burden on patients and their families, often leading to unnecessary tests and, in some cases, prolonged hospitalization. Early identification of citrin deficiency through NBS is associated with better outcomes compared to diagnoses made after symptoms have appeared [5]. The predictive scoring formula proposed in this study can reduce the false-negative rate and improve the sensitivity for detecting citrin deficiency, minimizing missed cases and facilitating an earlier diagnosis. As a result, early intervention and treatment can be implemented, improving overall clinical outcomes and alleviating the long-term burden on both the healthcare system and affected families.

At least 100 *SLC25A13* variations have been described, with differences in high-frequency regions of variations among different countries. In Japan, 11 mutations such as c.851_854del, c.1019_1177del, c.1231_1311del, and c.675C > A account for approximately 95% of the total mutations [35]. The hotspot mutation in Korea is IVS16ins3kb [36]. In our cohort, we found twenty-eight known and one novel *SLC25A13* variants, expanding the mutational spectra of CD. Previous studies have indicated that homozygous c.1177 + 1G > A or c.852_855delTATG mutations correlate with low birth height/weight, elevated transaminases, cholestasis, hypoproteinemia, and prolonged prothrombin time [12]. However, in our study, the distribution characteristics of gene mutations in both the “Newborn Screening” and “Missed Screening” groups were mostly represented by c.852_855del, IVS16ins3kb, and c.615 + 5G > A. This indicates that our data do not support a significant relationship between genotype and clinical manifestations or outcomes. It might be quite challenging to determine whether someone is a missed patient based solely on genetics. However, it is noteworthy that the frequency of c.1638_1660dup in the “Newborn Screening” group is significantly higher than in the “Missed Screening” group. Given the limited sample size, further validation is needed to establish the relationship between genotype and phenotype.

The present study has several strengths. First, we collected data on the characteristics of missed NICCD cases in Zhejiang Province, a high-incidence region, providing a theoretical basis for improving NICCD detection in other similar regions. Second, we identified and validated metabolites from MS/MS that can reduce the rate of missed screenings, offering a simple and feasible method. Third, we identified a gene mutation with a slight difference between missed and NBS-positive patients. However, our data only represented NICCD cases missed in Zhejiang Province. Future research could extend to multiple regions with both high and low NICCD incidence rates, with regular data collection (e.g., quarterly or annually) to create a time-series dataset. Using machine learning to dynamically adjust cut-off values based on different time periods and population characteristics may further improve NICCD detection.

In conclusion, we propose a novel predictive scoring formula incorporating levels of Cit, Gly, Phe, Orn, and C8, which demonstrates high sensitivity in identifying missed NICCD cases in our retrospective study. This method provides a simple and cost-effective approach to assist clinicians in the early detection of NICCD patients.

## Supplementary Information

Additional file 1

## Data Availability

The data supporting the findings of this study are available at https://doi.org/10.6084/m9.figshare.27628812.

## Abbreviations

Cit: Citrulline
Phe: Phenylalanine
Orn: Ornithine
Gly: Glycine
NICCD: Neonatal intrahepatic cholestasis caused by citrin deficiency
CD: Citrin deficiency
NBS: Newborn screening
DBS: Dried blood spots
AGC: Aspartate-glutamate carrier
ROC: Receiver operating characteristic
MS/MS: Tandem mass spectrometry
MCT: Medium-chain triglyceride

## References

[1] Tabata A, Sheng J-S, Ushikai M, Song Y-Z, Gao H-Z, Lu Y-B, et al. Identification of 13 novel mutations including a retrotransposal insertion in SLC25A13 gene and frequency of 30 mutations found in patients with citrin deficiency. J Hum Genet. 2008;53(6):534–45. 18392553 10.1007/s10038-008-0282-2

[2] Gu X, Han L, Yu Y. Current status and prospects of screening for newborn hereditary metaboolic disease. J Rare Dis. 2022;1(01):13–9.

[3] Zhang T, Zhu S, Miao H, Yang J, Shi Y, Yue Y, et al. Dynamic changes of metabolic characteristics in neonatal intrahepatic cholestasis caused by citrin deficiency. Front Mol Biosci. 2022;9: 939837. 36090036 10.3389/fmolb.2022.939837 PMC9449879

[4] Inui A, Ko JS, Chongsrisawat V, Sibal A, Hardikar W, Chang MH, et al. Update on the diagnosis and management of neonatal intrahepatic cholestasis caused by citrin deficiency: expert review on behalf of the Asian pan-pacific society for pediatric gastroenterology, hepatology, and nutrition. J Pediatr Gastroenterol Nutr. 2024;78(2):178–87. 38374571 10.1002/jpn3.12042

[5] Chen CY, Chang MH, Chen HL, Chien YH, Wu JF. The prognosis of citrin deficiency differs between early-identified newborn and later-onset symptomatic infants. Pediatr Res. 2023;94(3):1151–7. 37029238 10.1038/s41390-023-02585-3

[6] Hayasaka K. Pathogenesis and management of citrin deficiency. Intern Med. 2024;63(14):1977–86. 37952953 10.2169/internalmedicine.2595-23 PMC11309867

[7] Saheki T, Moriyama M, Funahashi A, Kuroda E. AGC2 (Citrin) deficiency-from recognition of the disease till construction of therapeutic procedures. Biomolecules. 2020. 10.3390/biom10081100. 32722104 10.3390/biom10081100 PMC7465890

[8] Hayasaka K, Numakura C, Yamakawa M, Mitsui T, Watanabe H, Haga H, et al. Medium-chain triglycerides supplement therapy with a low-carbohydrate formula can supply energy and enhance ammonia detoxification in the hepatocytes of patients with adult-onset type II citrullinemia. J Inherit Metab Dis. 2018;41(5):777–84. 29651749 10.1007/s10545-018-0176-1

[9] Hayasaka K, Numakura C. Adult-onset type II citrullinemia: Current insights and therapy. Appl Clin Genet. 2018;11:163–70. 30588060 10.2147/TACG.S162084 PMC6296197

[10] Chakravarthy MV, Pan Z, Zhu Y, Tordjman K, Schneider JG, Coleman T, et al. “New” hepatic fat activates PPARalpha to maintain glucose, lipid, and cholesterol homeostasis. Cell Metab. 2005;1(5):309–22. 16054078 10.1016/j.cmet.2005.04.002

[11] Tamamori A, Fujimoto A, Okano Y, Kobayashi K, Saheki T, Tagami Y, et al. Effects of citrin deficiency in the perinatal period: feasibility of newborn mass screening for citrin deficiency. Pediatr Res. 2004;56(4):608–14. 15295082 10.1203/01.PDR.0000139713.64264.BC

[12] Kido J, Haberle J, Sugawara K, Tanaka T, Nagao M, Sawada T, et al. Clinical manifestation and long-term outcome of citrin deficiency: report from a nationwide study in Japan. J Inherit Metab Dis. 2022;45(3):431–44. 35142380 10.1002/jimd.12483

[13] Lee NC, Chien YH, Kobayashi K, Saheki T, Chen HL, Chiu PC, et al. Time course of acylcarnitine elevation in neonatal intrahepatic cholestasis caused by citrin deficiency. J Inherit Metab Dis. 2006;29(4):551–5. 16736097 10.1007/s10545-006-0250-y

[14] Chen H-W, Chen H-L, Ni Y-H, Lee N-C, Chien Y-H, Hwu W-L, et al. Chubby face and the biochemical parameters for the early diagnosis of neonatal intrahepatic cholestasis caused by citrin deficiency. J Pediatr Gastroenterol Nutr. 2008;47(2):187–92. 18664871 10.1097/MPG.0b013e318162d96d

[15] Kobayashi K, Sinasac DS, Iijima M, Boright AP, Begum L, Lee JR, et al. The gene mutated in adult-onset type II citrullinaemia encodes a putative mitochondrial carrier protein. Nat Genet. 1999;22(2):159–63. 10369257 10.1038/9667

[16] Samuels DC, Song Y-Z, Zhang Z-H, Lin W-X, Zhao X-J, Deng M, et al. SLC25A13 Gene analysis in citrin deficiency: sixteen novel mutations in East Asian patients, and the mutation distribution in a large pediatric cohort in China. PLoS ONE. 2013;8(9): e74544. 24069319 10.1371/journal.pone.0074544 PMC3777997

[17] Lin WX, Zeng HS, Zhang ZH, Mao M, Zheng QQ, Zhao ST, et al. Molecular diagnosis of pediatric patients with citrin deficiency in China: SLC25A13 mutation spectrum and the geographic distribution. Sci Rep. 2016;6:29732. 27405544 10.1038/srep29732 PMC4942605

[18] Ohura T, Kobayashi K, Tazawa Y, Nishi I, Abukawa D, Sakamoto O, et al. Neonatal presentation of adult-onset type II citrullinemia. Hum Genet. 2001;108(2):87–90. 11281457 10.1007/s004390000448

[19] Tazawa Y, Kobayashi K, Ohura T, Abukawa D, Nishinomiya F, Hosoda Y, et al. Infantile cholestatic jaundice associated with adult-onset type II citrullinemia. J Pediatr. 2001;138(5):735–40. 11343052 10.1067/mpd.2001.113264

[20] Song YZ, Deng M, Chen FP, Wen F, Guo L, Cao SL, et al. Genotypic and phenotypic features of citrin deficiency: five-year experience in a Chinese pediatric center. Int J Mol Med. 2011;28(1):33–40. 21424115 10.3892/ijmm.2011.653

[21] Lin J, Lin W, Lin Y, Peng W, Zheng Z. Clinical and genetic analysis of 26 Chinese patients with neonatal intrahepatic cholestasis due to citrin deficiency. Clinica Chimica Acta. 2024. 10.1016/j.cca.2023.117617. 10.1016/j.cca.2023.117617 37890575

[22] Wang T, Ma J, Zhang Q, Gao A, Wang Q, Li H, et al. Expanded newborn screening for inborn errors of metabolism by tandem mass spectrometry in suzhou, china: disease spectrum, prevalence, genetic characteristics in a Chinese Population. Front Genet. 2019;10:1052. 31737040 10.3389/fgene.2019.01052 PMC6828960

[23] Streiner DL, Cairney J. What’s under the ROC? An introduction to receiver operating characteristics curves. Can J Psychiatry. 2007;52(2):121–8. 17375868 10.1177/070674370705200210

[24] Okano Y, Ohura T, Sakamoto O, Inui A. Current treatment for citrin deficiency during NICCD and adaptation/compensation stages: Strategy to prevent CTLN2. Mol Genet Metab. 2019;127(3):175–83. 31255436 10.1016/j.ymgme.2019.06.004

[25] Zeng H-S, Zhao S-T, Deng MEI, Zhang Z-H, Cai X-R, Chen F-P, et al. Inspissated bile syndrome in an infant with citrin deficiency and congenital anomalies of the biliary tract and esophagus: identification and pathogenicity analysis of a novel SLC25A13 mutation with incomplete penetrance. Int J Mol Med. 2014;34(5):1241–8. 25216257 10.3892/ijmm.2014.1929 PMC4199400

[26] Tang CF, Liu SC, Feng Y, Mei HF, Liu HP, Feng JW, et al. Newborn screening program and blood amino acid profiling in early neonates with citrin deficiency. Zhonghua Er Ke Za Zhi. 2019;57(10):797–01. 31594068 10.3760/cma.j.issn.0578-1310.2019.10.014

[27] Abuduxikuer K, Chen R, Wang Z-L, Wang J-S. Risk factors associated with mortality in neonatal intrahepatic cholestasis caused by citrin deficiency (NICCD) and clinical implications. BMC Pediatrics. 2019. 10.1186/s12887-018-1383-5. 30642297 10.1186/s12887-018-1383-5 PMC6330752

[28] Saheki T, Kobayashi K. Mitochondrial aspartate glutamate carrier (citrin) deficiency as the cause of adult-onset type II citrullinemia (CTLN2) and idiopathic neonatal hepatitis (NICCD). J Hum Genet. 2002;47(7):333. 12111366 10.1007/s100380200046

[29] Sinasac DS, Moriyama M, Jalil MA, Begum L, Li MX, Iijima M, Tsui LC. Slc25a13-knockout mice harbor metabolic deficits but fail to display hallmarks of adult-onset type II citrullinemia. Mol Cell Biol. 2004;24(2):527–36. 14701727 10.1128/MCB.24.2.527-536.2004 PMC343808

[30] Miyazaki T, Nagasaka H, Komatsu H, Inui A, Morioka I, Tsukahara H, et al. Serum amino acid profiling in citrin-deficient children exhibiting normal liver function during the apparently healthy period. JIMD Rep. 2019;43:53–61. 29654547 10.1007/8904_2018_99 PMC6323014

[31] Shigetomi H, Tanaka T, Nagao M, Tsutsumi H. Early detection and diagnosis of neonatal intrahepatic cholestasis caused by citrin deficiency missed by newborn screening using tandem mass spectrometry. Int J Neo Screen. 2018. 10.3390/ijns4010005. 10.3390/ijns4010005 PMC7548893 33072931

[32] Chen H-A, Hsu R-H, Chen Y-H, Hsu L-W, Chiang S-C, Lee N-C, et al. Improved diagnosis of citrin deficiency by newborn screening using a molecular second-tier test. Mol Genet Metab. 2022;136(4):330–6. 35798653 10.1016/j.ymgme.2022.06.007

[33] Lin Y, Liu Y, Zhu L, Le K, Shen Y, Yang C, et al. Combining newborn metabolic and genetic screening for neonatal intrahepatic cholestasis caused by citrin deficiency. J Inherit Metab Dis. 2019;43(3):467–77. 31845334 10.1002/jimd.12206

[34] Kido J, Häberle J, Tanaka T, Nagao M, Wada Y, Numakura C, et al. Improved sensitivity and specificity for citrin deficiency using selected amino acids and acylcarnitines in the newborn screening. Journal of Inherited Metabolic Disease. 2023. 10.1002/jimd.12673 37681292

[35] Kikuchi A, Arai-Ichinoi N, Sakamoto O, Matsubara Y, Saheki T, Kobayashi K, et al. Simple and rapid genetic testing for citrin deficiency by screening 11 prevalent mutations in SLC25A13. Mol Genet Metab. 2012;105(4):553–8. 22277121 10.1016/j.ymgme.2011.12.024

[36] Oh SH, Lee BH, Kim G-H, Choi J-H, Kim KM, Yoo H-W. Biochemical and molecular characteristics of citrin deficiency in Korean children. J Hum Genet. 2016;62(2):305–7. 27829683 10.1038/jhg.2016.131

