# Supplementary material for: Enhancing newborn screening sensitivity and specificity for missed NICCD using selected amino acids and acylcarnitines
Source: Orphanet J Rare Dis. 2025 Jan 11;20:17. doi: 10.1186/s13023-025-03532-7 (PMC11724517; doi:10.1186/s13023-025-03532-7)
Supplement: Supplementary file 1 — Additional file 1 [file 13023_2025_3532_MOESM1_ESM.docx]

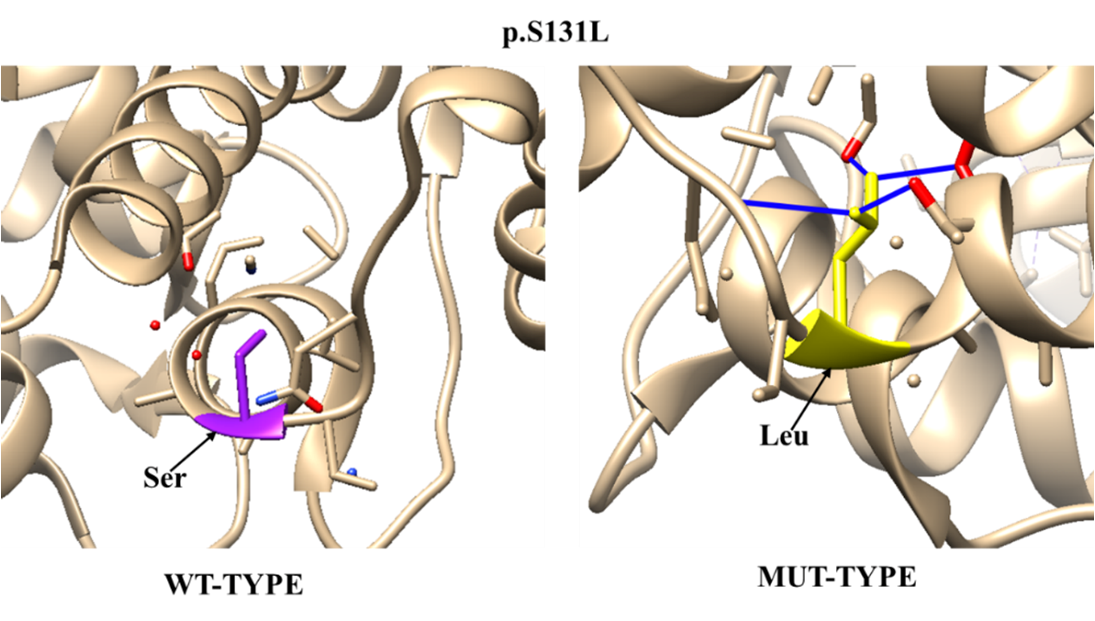


**Supplementary Figure** Structure stability analysis of the p.S131L variant on the partial tertiary structure of NICCD protein. Protein modeling showed that the wild-type amino-acid residue at 131-site colored purple has no contact with the amino-acid residue nearby. Blue lines show atom clash and contacts in the mutation type. Four atom clashes and contacts (colored blue) were found in this region (colored yellow) after Leucine substituted Serine, resulting in an unstable protein
